# Bromelain- and Silver Nanoparticle-Loaded Polycaprolactone/Chitosan Nanofibrous Dressings for Skin Wound Healing

**DOI:** 10.3390/gels9080672

**Published:** 2023-08-19

**Authors:** Yasaman Saghafi, Hadi Baharifar, Najmeh Najmoddin, Azadeh Asefnejad, Hassan Maleki, Sayed Mahmoud Sajjadi-Jazi, Alireza Bonkdar, Forough Shams, Kamyar Khoshnevisan

**Affiliations:** 1 Department of Biomedical Engineering, Science and Research Branch, Islamic Azad University, Tehran 1477893855, Iran; yasaman.saghafi90@gmail.com (Y.S.); najmoddin@srbiau.ac.ir (N.N.);; 2 Applied Biophotonics Research Center, Science and Research Branch, Islamic Azad University, Tehran 1477893855, Iran; 3 Research and Development Team, Evolution Wound Dressing (EWD) Startup Co., Tehran 1983963113, Iran; 4 Nano Drug Delivery Research Center, Health Technology Institute, Kermanshah University of Medical Sciences, Kermanshah 6715847141, Iran; 5 Cell Therapy and Regenerative Medicine Research Center, Endocrinology and Metabolism Molecular-Cellular Sciences Institute, Tehran University of Medical Sciences, Tehran 1411713137, Iran; 6 Medical Nanotechnology and Tissue Engineering Research Center, Shahid Beheshti University of Medical Sciences, Tehran 1983963113, Iran; 7 Department of Tissue Engineering and Applied Cell Sciences, School of Advanced Technologies in Medicine, Shahid Beheshti University of Medical Sciences, Tehran 1983963113, Iran; forough.shamss@gmail.com

**Keywords:** nanofibers, wound dressing, electrospinning, polycaprolactone, chitosan, silver nanoparticles, bromelain

## Abstract

A cutaneous wound is caused by various injuries in the skin, which can be wrapped with an efficient dressing. Electrospinning is a straightforward adjustable technique that quickly and continuously generates nanofibrous wound dressings containing antibacterial and anti-inflammatory agents to promote wound healing. The present study investigated the physicochemical and biological properties of bromelain (BRO)- and silver nanoparticle (Ag NPs)-loaded gel-based electrospun polycaprolactone/chitosan (PCL/CS) nanofibrous dressings for wound-healing applications. Electron microscopy results showed that the obtained nanofibers (NFs) had a uniform and homogeneous morphology without beads with an average diameter of 176 ± 63 nm. The FTIR (Fourier transform infrared) analysis exhibited the loading of the components. Moreover, adding BRO and Ag NPs increased the tensile strength of the NFs up to 4.59 MPa. BRO and Ag NPs did not significantly affect the hydrophilicity and toxicity of the obtained wound dressing; however, the antibacterial activity against *E. coli* and *S. aureus* bacteria was significantly improved. The in vivo study showed that the wound dressing containing BRO and Ag NPs improved the wound-healing process within one week compared to other groups. Therefore, gel-based PCL/CS nanofibrous dressings containing BRO and Ag NPs could be a promising solution for healing skin wounds.

## 1. Introduction

The skin acts as a natural protector against external pathogenic microbial, chemical, mechanical, and thermal stresses and can therefore be exposed to various injuries such as wounds, trauma, and burns [1,2]. The protection of body fluids, electrolytes, and nutritional components of the body depends on the safekeeping of the skin against severe physicochemical damage and microbial invasion. Tissue engineering, as a method to replace or improve portions of or whole biological tissues such as the skin, bladder, bone, blood vessels, and cartilage, holds great potential for the treatment of human diseases [3,4]. In recent years, a growing deal of attention has been given to promoting wound healing by suitable dressings. An ideal wound dressing should be applied to protect the wound against external contaminants and pathogenesis, as well as facilitate the healing process [5]. The most powerful features of a perfect dressing include adequate tensile strength, high biocompatibility, intense antibacterial activity, and sufficient permeability for the enhancement of humidity and oxygen [6,7,8]. Infection is another major problem that hinders wound healing and can cause various life-threatening complications, such as multiple organ system defects, bacteremia, and sepsis [9]. The primary approach to overcoming this problem is using wound dressings that contain antibacterial agents on the surface, which can gradually release the agents into the wounded area [10,11]. Effective antibacterial wound dressings have recently attracted a great deal of attention from researchers because they can inhibit the growth of Gram-positive and Gram-negative bacteria in the wound area [12].

Fibrous-containing bioactive agents are one of the eligible candidates for dressings employed for the promotion of wound healing. The range of the fibers’ diameter is from several micrometers down to a few nanometers. Their high surface area and high porosity subsequently resulted in effective controlled delivery of drugs and different biological molecules to the surrounding environment [10,11].

Researchers bestow a high value on natural polymers like chitin, chitosan (CS), and collagen due to their excellent antimicrobial and biological properties [13]. Innovative efforts have been made to produce nanofibrous dressings of multi-polymer mixtures—incredibly natural and synthetic polymer mixtures—for environmental use. In this case, most studies have investigated CS and polycaprolactone (PCL) for the fabrication of films, membranes [14], and electrospinning fibers [15,16]; however, a few studies have considered mixtures of these [17]. A one-layer wound dressing cannot meet all clinical needs at the same time because it does not have the characteristics of an ideal dressing, including surface wettability, antibacterial and anti-inflammatory activity, biodegradability, and biocompatibility [18]. Thus, bilayer dressings comprising two layers with various properties have received significant attention [19]. A high-density upper layer can protect the wound from infection and mechanical stress as well as prevent wound dehydration, and it can also provide a humid environment in the wound region [20]. Subsequently, the sub-layers come into contact directly with the wound area and should mimic the ECM structure to facilitate cell adherence and accelerate cell proliferation [21].

Among the biomaterials applied in manufacturing, CS, as an antibacterial wound dressing, significantly improves the antibacterial activity against Gram-positive and Gram-negative bacteria and limits infection in the wound area [21,22]. Also, it has been reported that positively charged CS interacts with negatively charged microbial cell membranes [23]. The negative charge of lipopolysaccharides present in Gram-negative bacteria membranes engages in an ionic type of binding with the positively charged amino groups of CS. Consequently, this interaction leads to impeded nutrient flow and eventual bacterial death [22]. Additionally, CS-based wound dressings provide positive effects on all wound-healing stages, such as the synthesis of hyaluronic acid and type II collagen [24]. CS is a biocompatible, biodegradable, and non-toxic polysaccharide with excellent properties, including adhesive capability and antimicrobial/antifungal activity [25]. Despite these benefits, it has drawbacks such as low dissolvability in water, poor mechanical efficiency, and poor dispensability consistency [26].

PCL is a common synthetic polymer in biomedical applications owing to its biocompatibility, safety, and resistance. Although electrospun PCL webs are structurally similar to the ECM in living tissues, their hydrophobicity causes flow cell loading and inhibits cell adhesion, proliferation, and differentiation [26,27]. PCL’s hydrophobicity should be reduced to improve its biological properties. Further, PCL blending with other hydrophilic polymers can provide a fruitful approach due to enhancing the overall material properties [28,29]. Correspondingly, apart from highly required biological properties, the dressing should also have the appropriate mechanical properties depending on the tissue, which has been proposed with the highly mechanical properties of PCL [30].

CS provides moderate antimicrobial activity due to its cationic excess. The antibacterial activity of silver species has been well known since ancient times [31]. Studies have shown that a double-layer wound dressing consisting of silver nanoparticles (Ag NPs) and CS can significantly accelerate the healing rate of skin wounds [32]. The antibacterial and antifungal behavior of biosynthesized Ag NPs against various microorganisms has been widely noted [33]. Unlike antibiotics, Ag NPs are released after exposure to microbes and affect microorganisms [34]. In addition, other essential features of these NPs include their being non-toxic, biocompatible, heat-resistant, and very highly durable and their failure to create and increase resistance and compatibility in microorganisms. However, some researchers have suggested that blending CS and PCL can improve the disadvantages of CS, for instance, its spin ability and mechanical strength, during the electrospinning process [32,33]. The blending of PCL/CS introduced the advancement of dressing properties concerning an acceptable degradation rate, water retention, and vascularization during the wound-healing process. Thus, the idea of adding any additional layer of a suitable biopolymer onto CS/PCL matrices has been recommended to enhance PCL/CS’s admissibility as a skin dressing [34,35].

Bromelain (BRO) is a major sulfhydryl proteolytic enzyme present in pineapple kernels and stems. This enzyme consists of a mixture of different thiol endopeptidases and non-protease components [36]. Studies have shown that BRO has multifarious therapeutic attributes, e.g., anti-oxidant, anti-inflammatory, immunomodulatory, anti-thrombotic, cardioprotective, fibrinolytic, and anti-cancer properties [37]. Hence, it has contributed to the treatment and relief of various diseases, such as angina pectoris, bronchitis, sinusitis, surgical trauma, osteoarthritis, diarrhea, and dermatological issues [38]. Furthermore, it has shown potential wound-healing activity by the efficient debridement of necrotic tissue and the modulation of inflammatory reactions [39].

Various biomimetic systems have been produced in recent years, comprising microporous gels and substrates with varying chemistry and topography. The ideal substrate for wound-healing applications should be biocompatible, biodegradable, and capable of supporting cell development in a manner comparable to the in vivo microenvironment. Although the effectiveness of microporous scaffolds was proved in some applications, they are not an actual replica of an ECM structure, because they influence cell binding. In addition, the bulk of ECM proteins are fibrous; however, nanofibrous dressings are more bio-mimicking [40]. Nanofibers (NFs) are principally interesting thanks to their simplicity of fabrication, high surface area to volume ratio, diversity of composition, customizable shape and physicochemical characteristics, bioactive compound loading potential, programmable release, and degradation kinetics [41,42].

Many natural and artificial polymers have been electrospun to create a three-dimensional (3D) ECM that resembles NFs [43]. Some recent research has suggested using merged PCL and CS for NF production due to the mechanical strength, processability, and biocompatibility of PCL and the ECM mimicking qualities of CS [44,45]. It is believed that CS-based dressings have been widely applied as wound dressings for the promotion of wound healing [46,47].

In the present study, biocompatible gel-based PCL/CS nanofibrous scaffolds were prepared using novel 3-nozzle electrospinning technology and then coated with Ag NPs and BRO by sprinkling these on the surface of the electrospun NFs. The present study aimed to investigate the synergic effects of Ag NPs and BRO on the antibacterial properties of composite NFs. The resultant physicochemical and in vitro properties in the presence and absence of an Ag NP and BRO coating layer on the wound dressing were scrutinized. An animal study containing a control, PCL/CS, and PCL/CS along with BRO and Ag NPs was conducted to exhibit the functionality of the developed products as a skin wound dressing.

## 2. Results and Discussion

### 2.1. Characterizations of Ag NPs

**Figure 1:**
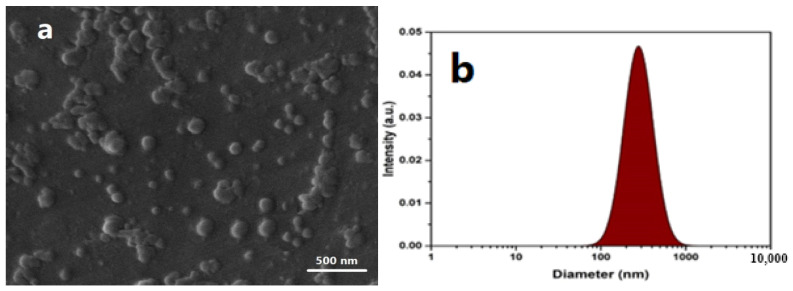
Scanning electron micrograph of Ag NPs at 20 kV (**a**) and hydrodynamic diameter and dispersity of the particles (**b**). In DLS analysis, the sample was used without dilution and was performed in triplicate.

The diameter and shapes of Ag NPs are shown in Figure 1. In the SEM image, the average size of the NPs was attained at about 55 nm (Figure 1a). The particles’ hydrodynamic size and distribution were examined by Particle Size Analyzer (VASCO, CORDOUAN, France). The particles are monodispersed, and their hydrodynamic size was about 135 nm, as represented in Figure 1b. DLS analysis requires a larger number of NPs compared to SEM, and it therefore provides significantly better statistics. However, in most cases, the hydrodynamic particle size obtained by DLS was larger than the values obtained by SEM [31].

### 2.2. Nanofibrous Scaffold Characterizations

**Figure 2:**
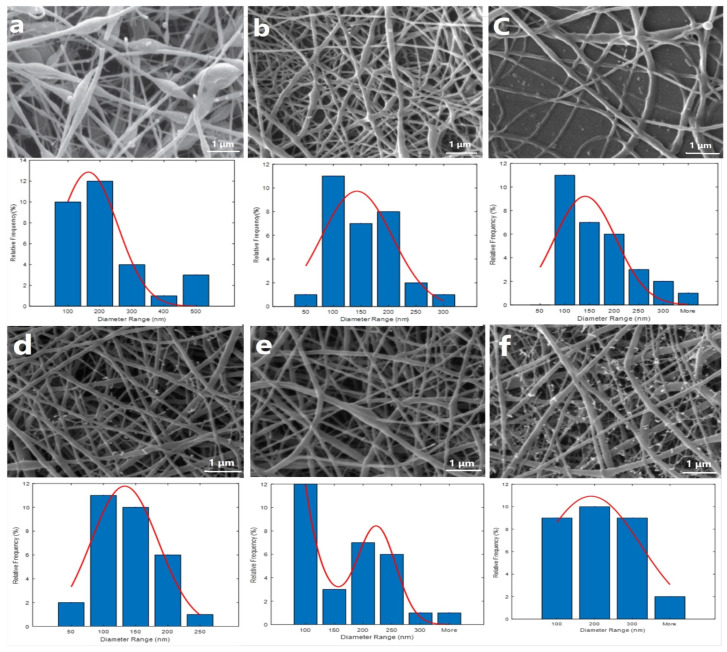
SEM micrographs of NFs show the morphology and random distribution of fibers: nanofibrous webs of PCL/CS with ratios of (**a**) 50:50, (**b**) 60:40, (**c**) 70:30, and (**d**) 60:40 containing Ag NPs, (**e**) 60:40 containing Bro, and (**f**) 60:40 containing Ag NPs + Bro. All samples were coated with a thin layer of gold to produce a conductive surface and were examined at 15 kV.

NFs’ shape, diameter, and morphology were observed and measured using SEM (Figure 2). Different polymer (i.e., PCL/Cs) ratios were investigated to obtain a consistent bead-less nanofibrous structure (Table 1 and Figure 2).

The SEM micrographs of the PCL/CS (50-50) (average diameter 83 ± 27 nm) NFs exhibited spindle-like beads (Figure 2a). By increasing the PCL content (i.e., 60:40 PCL/CS), a uniform mesh and fine NFs with 126 ± 8 nm average diameter were acquired (Figure 2b). Further increasing the PCL ratio (70:30 PCL/CS) resulted in fibers with finer diameters (average diameter of 145 ± 73 nm); however, the chain entanglement was dropped, and the solution jet was broken during elongation and evaporation of the solvent (Figure 2c). Therefore, the 60:40 ratio was applied as an optimum mixture for further modification. Figure 2d shows the SEM image of PCL/CS containing Ag NPs. As detailed in the image above, the uniformity of the scaffolds increased, and a bead-less fiber was obtained (113 nm) [45]. The presence of conductive agents including Ag NPs produced enhanced electric charges carried out by the electrospinning jet [46], so the fiber diameter dropped significantly [45]. The SEM image showed white grains that may indicate the presence of NPs in the substrate. By adding Bro, the fiber diameter was changed (i.e., 147 ± 16 nm), as shown in Figure 2e. The average diameter of the NFs along with both Ag NPs and Bro was obtained as 185 ± 24 nm, which is in the range of ECM collagen fibers (50 to 500 nm). Thus, it can be suggested that the produced nanofibers may mimic some structural features of the ECM and provide an environment for cell adhesion, proliferation, differentiation, and improved skin tissue regeneration at the wound site [48]. Figure 2f shows the SEM image of PCL/CS containing 60:40 Ag NPs + Bro (average diameter 176 ± 63 nm).

### 2.3. Fourier Transform Infrared Analysis

**Figure 3:**
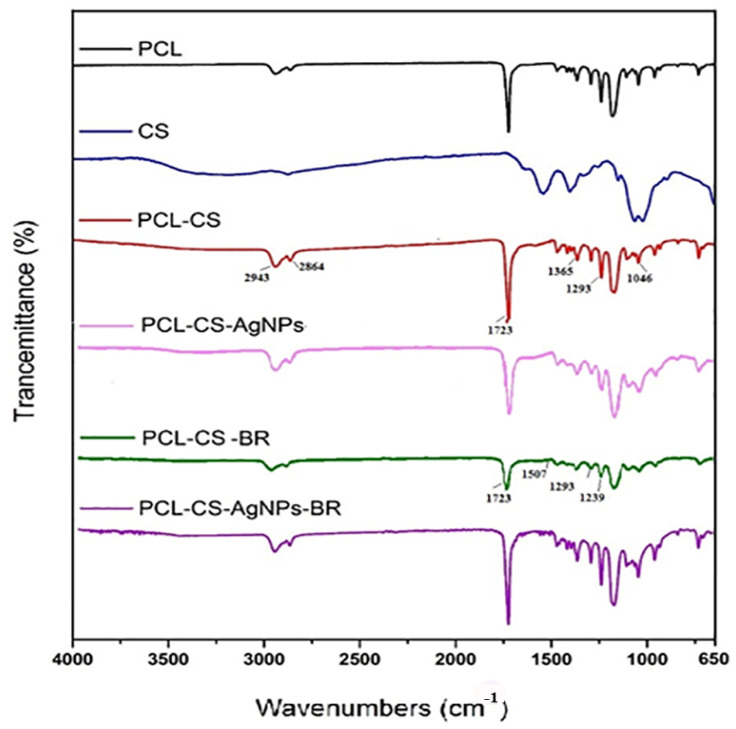
FTIR spectra of the gel-based PCL/CS, PCL/CS-BRO, PCL/CS-Ag NPs, and PCL/CS-BRO-Ag NPs scaffolds. For the FTIR, 1 mg of the samples was mixed with 300 mg of KBr under a vacuum. Then, IR spectra were recorded in the range of 400 and 4000 cm^−1^.

The IR spectra of all samples (CS/PCL, CS/PCL-Ag NPs, CS/PCL-BRO, and CS/PCL-Ag NPs-BRO) in Figure 3 confirmed the successful combination of CS and PCL in the electrospun NFs. Specific characteristic peaks at certain wavenumbers indicated the presence of PCL in the NFs (1723 cm^−1^, 1293 cm^−1^, and 1365 cm^−1^). The spectra revealed the presence of CS, with characteristic peaks at 2864 cm^−1^, 2943 cm^−1^, and 1046 cm^−1^, as well as a peak at 1723 cm^−1^ showing carboxylate adsorption [49,50,51].

In the FTIR spectrum of the PCL/CS/BRO scaffold, specific IR bands at 1239 cm^−1^ and 1293 cm^−1^ indicated the presence of guanidinium monoalkyl, and the peak at 1723 cm^−1^ confirmed the group C = O and the presence of amino acids like asparagine and glutamine [52]. For both PCL/CS-Ag NPs and PCL/CS-BRO-Ag NPs, the characteristic peaks observed at 3000–3650 cm^−1^ were attributed to symmetric vibrations of OH and NH. These peaks indicated interactions between Ag NPs and the OH and NH groups of CS, with the interaction decreasing as the amount of Ag NPs increased [53,54].

### 2.4. Mechanical Measurement

**Figure 4:**
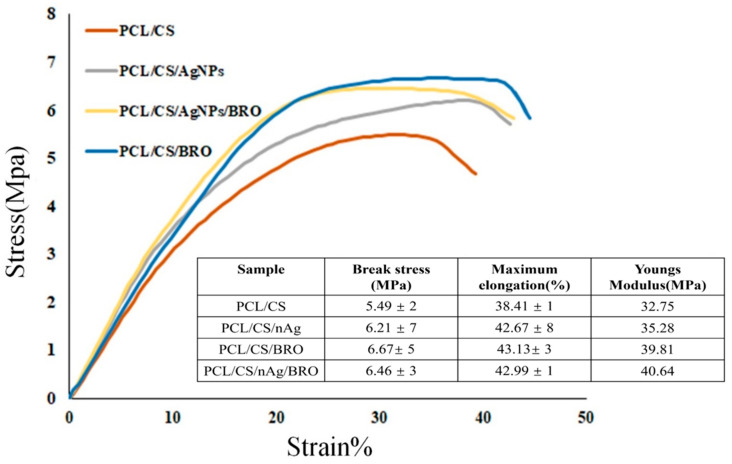
The tensile stress–strain curves of gel-based electrospun nanofibrous scaffolds along with the related parameters. The samples were cut into 2 × 5 cm^2^ pieces and were applied to the jaws at 1 mm/min and 500 N load cell. The test was performed three times for each sample, and the values were reported as mean ± standard deviation (SD).

The tensile strength results of the obtained scaffolds (Figure 4) revealed that adding Ag NPs and BRO increased the tensile stress–strain curves due to chemical bonding with the base polymers [45]. The proposed NFs had even better tensile strength, attributed to the higher crosslinking of BRO with CS (2% *w*/*v*) compared to NFs with Ag NPs or both BRO and Ag NPs [55].

### 2.5. Contact Angle of NFs

**Figure 5:**
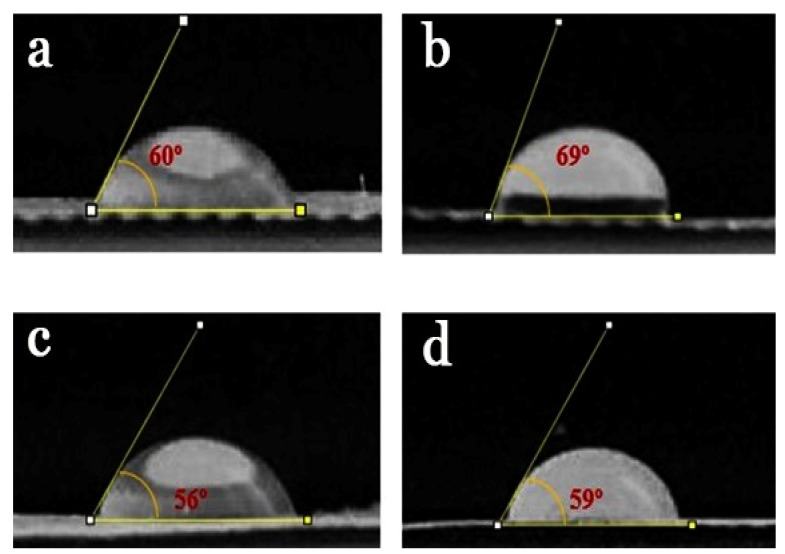
Contact angle images of PCL/CS (**a**), PCL/CS-BRO (**b**), PCL/CS-Ag NPs (**c**), PCL/CS-BRO-Ag NPs (**d**) NFs. The test dressings were cut to 15 × 20 mm^2^, and a drop of distilled water (2 µL) was spilled on each sample surface. The droplet figure was photographed after 3 s.

The hydrophilicity of nanofibrous dressings is a central factor for biocompatibility and cellular interactions such as cell adhesion, differentiation, and proliferation [48,56]. The contact angle measurement results obtained from gel-based PCL/CS (a), PCL/CS-BRO (b), PCL/CS-Ag NPs (c), and PCL/CS-Ag NPs-BRO (d) NFs were recorded after 3 s (Figure 5). The PCL/CS NFs displayed inherent hydrophilicity with a contact angle of 60° due to the presence of CS as a rather hydrophilic polymer [53] (Figure 5a). As shown in Figure 5b, the PCL/CS-BRO NFs exposed a contact angle of 69°, which proves hydrophobic interaction due to non-polar chain amino acids in the BRO structure [54]. On the contrary, according to Figure 5c, by adding Ag NPs, the hydrophilicity of NFs (56°) improved due to an increase in crosslinking with the polymer structure. Similarly, the findings were consistent with the background hypothesis presented by Li and colleagues [57], who stated that Ag NPs might enhance membrane surface hydrophilicity. Following Figure 5d, where both Ag NPs and BRO are presented, the contact angle increased, and the hydrophilicity decreased. Therefore, adding both BRO and Ag NPs did not have much of an effect on the hydrophilicity and hydrophobicity of the initial obtained NFs. All samples have a contact angle between 40 and 70°, which is suitable for cell attachment and proliferation.

### 2.6. BRO Activity

The results of an azocasein test indicated that upon adding bromelain to the nanofiber structure, its activity reached 87% of the initial value. This effect may be attributed to the electrospinning process as well as a possible disruption in the enzyme’s release from the fiber structure [36,37].

### 2.7. Antibacterial Results

**Figure 6:**
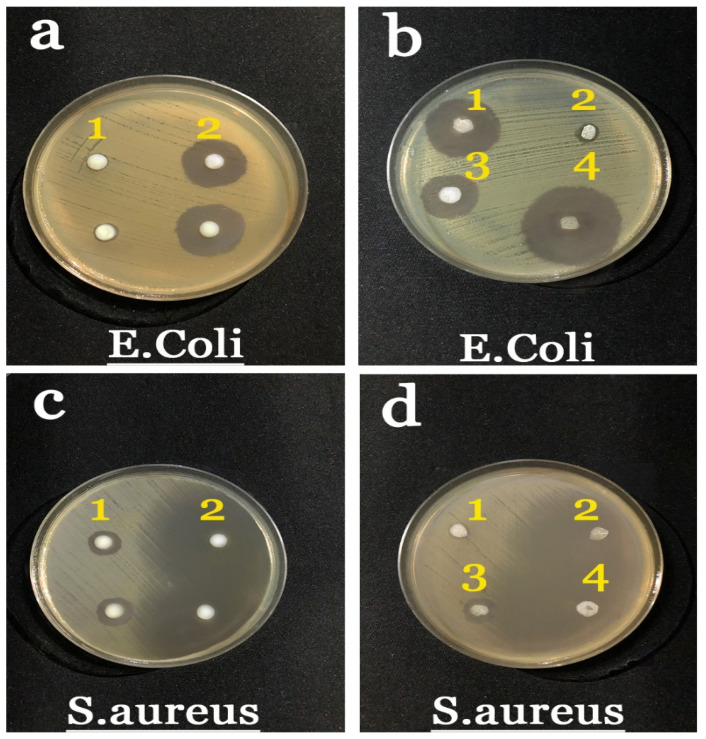
Antibacterial susceptibility by the zone inhibition against *E. coli* (**a**,**b**) and *S. aureus* (**c**,**d**): free BRO (1a, 1c), free Ag NPs (2a, 2c), PCL/CS (2b, 1d), PCL/CS-Ag NPs (1b, 2d), PCL/CS-BRO (3b, 3d), and PCL/CS-Ag NPs-BRO (4b, 4d).

The antibacterial effects of electrospinning the NFs, BRO solution, and Ag NPs were determined against *Escherichia coli* (*E. coli*) (ATCC 25922) and *Staphylococcus aureus* (*S. aureus*) (ATCC 25923). The results of the antimicrobial test are displayed in Figure 6. According to the plate images, sample 2b, 1d, which is a gel-based PCL/CS NF, was not observed with a significant inhibition zone and did not provide inhibitory activity on the growth of both bacterial strains. Although CS’s antibacterial activity has been proven in many studies [56], its antibacterial activity is affected by the molecular weight, degree of deacetylation, and concentration [58]. It was observed that (1b, 2d) Ag NPs exhibited the largest inhibition zone at about 24 ± 2 and 19 ± 1 mm against *E. coli* and *S. aureus*, respectively. Based on the articles and conducted experiments, the presence of Ag NPs provided antibacterial properties in the obtained NFs, because bacteria are affected by silver ions via their release into the bacterial cell wall, where they then destroy the bacteria [53,59]. Moreover, the ions can remove the bacterial respiratory enzyme that helps bacteria survive. It can be noted that the reduced size of Ag NPs renders a significant effect on the antibacterial property [53,60,61].

In the sample 3b, 3d, which contained BRO, antibacterial activity with an inhibition zone was attained around 10 ± 2 mm on *E. coli*; however, the sample did not provide high inhibitory activity against *S. aureus* (6 ± 1 mm). The suppression of growth and growth inhibition of both strains was observed in the samples (4b, 4d). The obtained results revealed that the largest inhibition zone reached about 25 ± 2 and 12 ± 1 mm against *E. coli* and *S. aureus*, respectively.

Overall, the antibacterial activity of NFs against *S. aureus* was higher than that against *E. coli*. This observation is due to the fact that the cell wall structure of these bacteria is dissimilar. The outer membrane of Gram-negative bacteria contains lipopolysaccharides and phospholipids in its outer and inner cortex, respectively; however, Gram-positive bacteria do not contain a lipopolysaccharide layer.

### 2.8. MTT Assay Results

**Figure 7:**
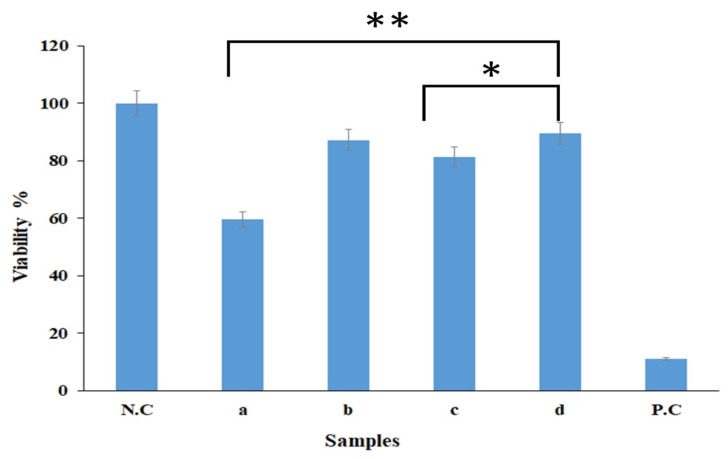
Cell viability by the MTT assay: (N.C) negative control, (a) PCL/CS, (b) PCL/CS-Ag NPs, (c) PCL/CS-BRO, and (d) PCL/CS-BRO-Ag NPs, (P.C) positive control after 24 h. * *p* ˂ 0.05 and ** *p* ˂ 0.01 using Mann–Whitney U test.

The in vitro viability activities of samples were assessed by MTT assay for 24 h. According to Figure 7, The sample containing gel-based PCL/CS NFs showed high cytotoxicity in mouse fibroblast cells within 24 h (Table 2). BRO NPs displayed higher cytotoxicity than that of Ag NPs. This is owing to the fact that Ag NPs can cause adverse biological effects such as cytotoxicity and genotoxicity effects, which are dose-dependent [32]. In several studies, the use of low concentrations of Ag NPs has been investigated for high survival and cell proliferation [59,62]. PCL/CS-Ag NPs-BRO NFs improved cell viability after 24 h because of the positive effect of Ag NPs and BRO on the gel-based PCL/CS NFs. The degree of cytotoxicity is negligible and close to that of the control sample. Thus, the proposed PCL/CS-Ag NPs-BRO mat could be applied as a suitable structure for cell adhesion, growth, and migration.

### 2.9. Animal Study

**Figure 8:**
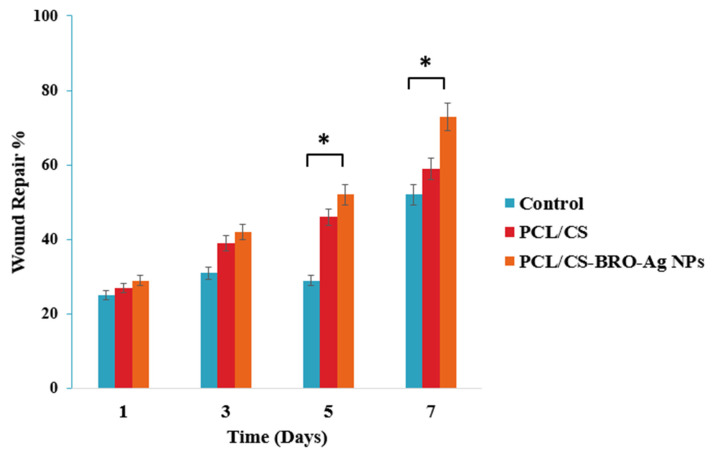
Wound repair % of 2nd degree burn rat model on different days. * *p* ˂ 0.05 using ANOVA test.

The results of the healing process from a second-degree burn model indicated that there was no significant difference between the groups treated with wound dressings and the control group on the first and third days (Figure 8). However, on the fifth day, both samples treated with wound dressings, namely, PCL/CS and PCL/CS-BRO-Ag NPs, exhibited a significant difference (*p* < 0.05) compared to the control, indicating an acceleration of the healing process. By the seventh day, the rate of wound healing in the control sample and the sample containing the PCL/CS wound dressing was similar. However, the group treated with NFs containing bromelain and Ag NPs showed a significant difference compared to the other groups. The presence of Ag NPs reduces the probability of infection at the wound site.

**Figure 9:**
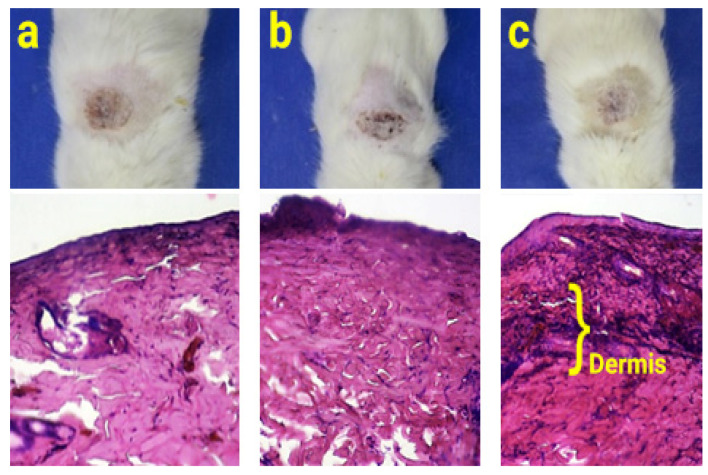
Rat skin wound repair and related H&E staining of wound-healing histology in different groups after 7 days: (**a**) control, (**b**) PCL/CS, (**c**) PCL/CS-BRO-Ag NPs.

Furthermore, Ag NPs effectively transformed fibroblasts into myofibroblasts, facilitating quick wound closure and promoting the proliferation and migration of keratinocytes [63,64]. Bromelain also reduces inflammation and swelling by suppressing kinin production, thereby accelerating wound healing [65]. Figure 9 shows that after 7 days, the skin layers in the group treated with PCL/CS-BRO-Ag NPs are more obviously formed, and the dermis layer is visible.

## 3. Conclusions

We evaluated the preparation, characteristics, and antibacterial and cell viability properties of a gel-based electrospun PCL/CS dressing co-loaded with Bro and Ag NPs to promote wound healing. The optimized electrospun PCL/CS (60:40) nanofibrous mat was co-loaded with BRO and Ag NPs through the electrospray method, which led to the formation of homogenous NFs with a nano-scale diameter and uniform morphology. The relevant analyses specified the successful loading and structural stability of the components. Also, the results indicated the high mechanical strength and suitable hydrophilicity of the prepared mats. The gel-based PCL/CS-Ag NPs-BRO nanofibrous dressing provided remarkable antibacterial activities against both Gram-positive and -negative bacteria. Moreover, the dermal fibroblast viability measurement confirmed the mat’s high biocompatibility and proper cell proliferation effects. The animal study displayed that the obtained mat enhanced the speed of wound healing over a week. Therefore, gel-based PCL/CS nanofibrous wound dressings co-loaded with BRO and Ag NPs can be exploited as an efficient scaffold for skin regeneration and wound-healing applications.

## 4. Materials and Methods

### 4.1. Materials

Chitosan (medium molecular weight, DD of 85%) and polycaprolactone (average Mn 80 kDa) were purchased from Sigma Aldrich, St. Louis, MO, USA. Formic acid, acetic acid, trisodium citrate, and silver nitrate were purchased from Merck Millipore, Darmstadt, Germany. Bromelain was purchased from Biozym, Hamburg, Germany, and other materials were provided from authentic centers unless otherwise specified.

### 4.2. Synthesis of Ag NPs

A simple reduction method was applied to fabricate Ag NPs [42]. Briefly, trisodium citrate (1% *w*/*v*) and silver nitrate (0.02% *w*/*v*) solutions were prepared in distilled water. Then, the silver nitrate solution was heated to boiling temperature using a magnetic heater stirrer. After that, the trisodium citrate solution was added dropwise to the boiling silver nitrate until the solution turned colored (pale yellowish-brown). Then, the heating was stopped, but dispersing was started by raising the stiller round. Then, the solution was placed on another stiller with a temperature of 25 °C for 15 min at 1000 rpm. The NPs obtained were stored in amber bottles at 4 °C. The formed colloidal Ag NPs were characterized and measured until day 14 after synthesis by DLS and SEM. These NPs were the reference Ag NPs for the study.

### 4.3. Fabrication of Free PCL/CS NFs

The fabrication of gel-based PCL/CS NFs started by mixing PCL (14% *w*/*v*) and CS (2% *w*/*v*) in a 3:1 ratio of formic acid: acetic acid solvent. After 2 h, the mix of CS and PCL with different blending ratios (Table 1) was kept on the magnetic stirrer at 250 rpm for 30 min. Then, the solution was electrospun under ambient conditions (a temperature of 22 to 25 °C and relative humidity of 45% to 52%) at a flow rate of 100 mL/h and with a 10 cm distance between the needle and collector, under 16 kV electrical potential.

### 4.4. Fabrication of PCL/CS-Ag NPs-BRO Mat

**Figure 10:**
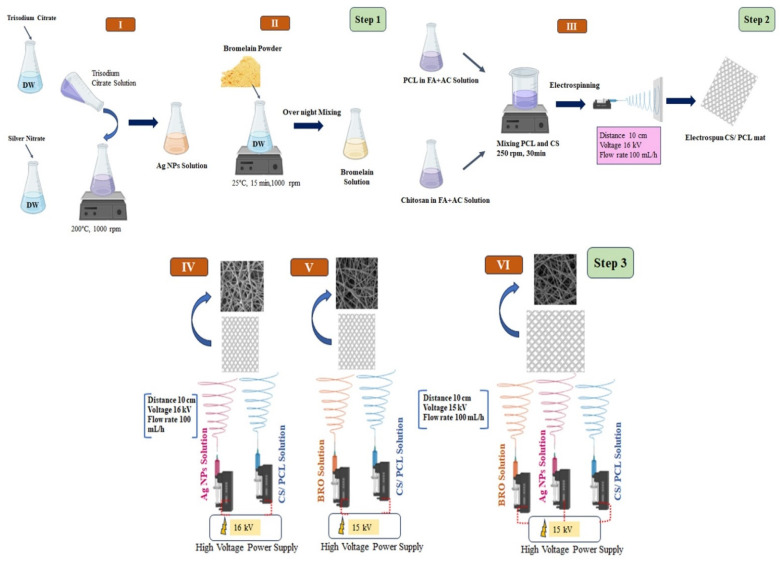
Fabrication processes of electrospinning. Step 1: Fabrication of Ag NPs (**I**) and BRO (**II**), step 2: fabrication of PCL/CS NFs (**III**), step 3: fabrication of PCL/CS-Ag NPs (**IV**), PCL/CS-BRO (**V**), and PCL/CS-Ag NPs-BRO (**VI**) scaffolds.

NFs were fabricated by electrospinning technique at room temperature. In the first step, the optimal polymer solution PCL/CS (60:40) (by mixing 14 % PCL *w*/*v* and 2% CS *w*/*v* in a 3:1 formic acid: acetic acid solvent system) was filled in a 1 mL syringe and equipped with a 29G needle. In addition, 1% *w*/*v* of Ag NPs and 2% *w*/*v* of BRO were prepared separately and then placed in individual syringes for the electrospinning process. In the next step, according to Figure 8, for fabrication of PCL/CS NFs including Ag NPs and BRO, the solutions of Ag NPs and BRO were separately electrosprayed on the surface of the base NFs. The solutions were drawn from the syringes with a flow rate of 0.1 mL/h using a syringe pump and were spun at a 10 cm distance between the needle and collector plate, under an electrical field of 16 kV potential. These processing parameters were standardized after several variations to obtain NFs of preferred quality. The resultant wound dressings (PCL/CS, PCL/CS-Ag NPs, PCL/CS-BRO, and PCL/CS-Ag NPs-BRO) were collected on 5 × 5 cm^2^ drum that was pre-adhered to aluminum foil for further analysis according to Figure 10.

### 4.5. Washing and Sterilization of Nanofibrous Dressing

Before performing the extraction procedure, all attained gel-based NFs, including PCL/CS, PCL/CS-Ag NPs, PCL/CS-BRO, and PCL/CS-BRO-Ag NPs mats, were washed at least three times with distilled water to ensure that the free materials were removed. Then, they were sterilized under a UV lamp (265 nm) for 4 h in a laminar flow cabinet (Pasteur Institute of Iran Class II Biological Safety Cabinet, Tehran, Iran) to keep the structural properties of the NFs intact.

### 4.6. Characterization of Nanofibrous Dressing

#### 4.6.1. Morphology Observation

The morphology of all nanofiber scaffolds was observed by a scanning electron microscope (SEM) (AIS2100, SERON TECHNOLOGIES, Anseong, Korea). In this regard, samples were coated with a thin layer of gold to produce a conductive surface and reduce charging.

#### 4.6.2. Fourier Transform Infrared Spectroscopy

The chemical characteristics of raw materials and the structures of samples were evaluated using Fourier transform infrared (FTIR) spectra (Nicolet Magna-IR 560) by the KBr method. As such, 1 mg of the materials and samples were mixed with 300 mg of KBr under a vacuum. Then, IR spectra were recorded in the range of 400 to 4000 cm^−1^.

#### 4.6.3. Tensile Testing

Tensile tests of the nanofiber membranes were carried out with a Universal Testing Machine (Instron TM—SM, England). The samples were cut into a rectangular shape with equal sizes of 2 × 5 cm^2^ and then placed in the middle of a paper frame with dimensions of 5 × 5 cm^2^ to prevent sample damage during the study. The jaws were spaced at a 1 mm/min speed by applying a 500 N load cell (according to the ASTM D 882 standard) until rupture occurred, and the stress–strain curve was plotted. The average values of tensile properties were achieved from the results of the four tests and expressed as the mean ± standard deviation (SD).

#### 4.6.4. Contact Angle Determination Test

All gel-based NFs were cut to 15 × 20 mm^2^ and were placed on a retentive base. A drop of distilled water (2 µL) was spilled onto each sample surface. The droplet image was photographed after 3 s, and the contact angle was gauged by ImageJ software.

#### 4.6.5. BRO Activity

Enzyme activity was measured using the azocasein method (REF). Briefly, a 1.0% *w*/*v* solution of azocasein was prepared in 0.2 M Tris-HCL and 1 mM calcium chloride. The pH of the solution was then adjusted to 7.2. The enzyme activity was measured both before and after combination into the fiber structure. The test was conducted in triplicate at 4 °C.

#### 4.6.6. Inhibition Zone Assay (Antibacterial Activity Measurements)

The antibacterial properties of the samples were evaluated according to the ISO 20645: 2004 standard Disc diffusion method. The samples were tested against *E. coli* (ATCC 25922) and *S. aureus* (ATCC 25923) as a model for Gram-negative and Gram-positive bacteria, respectively [49]. One hundred milliliters of *E. coli* and *S. aureus* were cultured on Luria–Bertani (LB) agar plates used for the antibacterial activity tests. Gel-based PCL/CS NFs were used as the control samples. The PCL/CS containing Ag NPs, BRO, and Ag NPs/BRO were applied as the test samples. The obtained dressings were cut into small circular pieces (5 mm) and placed over the solidified agar gel. Then, the plates were incubated for 24 h at 37 °C in a bacteriological incubator. Subsequently, the zone of bacterial inhibition was monitored.

#### 4.6.7. Cytotoxicity Test (MTT Assay)

An MTT assay was carried out to determine the potential cytotoxicity and viability of fibroblast cells involved in cutaneous wound healing [66]. The L929 cell (NCBI C161) (Pasteur Institute, Iran) was exploited for the proliferation study. After defrosting the cells, they were moved to a flask containing DMEM medium with 10% FBS, and then the flask was incubated at 37 °C and 5% carbon dioxide [44]. The extraction process of the sterilized samples was carried out according to the ISO 10993-5:2009 standard.

All samples were cut into circular pieces and sterilized by exposing them to UV light at 254 nm for 4 h. Subsequently, the samples were positioned at the bottom of the wells in a 96-well microplate using sterilized silicon rubber washers. A total of 10,000 cells in 100 μL of DMEM containing 10% FBS were added to each well. The entire plate was then incubated at 37 °C for 24 h. Then, the culture medium was removed, and 100 μL of MTT solution (0.5 mg/mL) was added to each well, followed by incubation in the dark for 4 h. Finally, the MTT solution was removed, and 50 μL of isopropanol was added to dissolve the purple formazan crystals. The plate was placed on a shaker for 15 min. All assays were carried out in triplicate. The same method was applied to the control group, but the cells were not treated with the samples. Then, the absorption of each well was read out by a microplate reader (STAT FAX 2100, USA) at 545 nm. The cell viability of the samples was calculated using Equation (1):

% Cell viability = (Mean OD of sample/Mean OD of control) × 100(1)

#### 4.6.8. Animal Study

Male Wistar rats weighing approximately 250 g was used for the animal study. The animal experiments were carried out following the guidelines established by the Ethic Committee for Animal Experiments at Science and Research Branch, Islamic Azad University (Ethical code number: IR.IAU.SRB.REC.1401.235). The studies were also in accordance with the EU Directive 2010/63/EU for animal experiments and any subsequent amendments, or similar ethical standards. The animals were housed in separate cages with a 12 h light and 12 h dark cycle and had unrestricted access to food and water. To establish a burn model, the rats were randomly divided into three groups, each consisting of 5 rats. One group served as the control, another was treated with PCL/CS NFs, and the third received the PCL/CS-BRO-Ag NP wound dressing. Subsequently, the rats were anaesthetized by intraperitoneal injection of ketamine (80 mg/kg) and xylazine (10 mg/kg), and the hair on their backs was shaved. A circular metal piece with a 1 cm diameter was immersed in boiling water to induce second-degree burns. After 5 min, it was placed on the rats’ back skin for 10 s [67].

The day of the burning procedure was designated as day zero. On the 3rd, 5th, and 7th days following the burns, images of the wounds were captured, and the wound area was calculated using image analysis software (ImageJ). The healing rate of the wounds was determined using Equation (2). On the seventh day, the skin at the wound site was excised, fixed in 10% formalin, and used to prepare a pathological slide. Hematoxylin-eosin staining was employed to examine the formation process of different skin layers.

(2) $$Wound\;repair\;\%=(W_{0}-W_{1})/W_{0}\times 100$$

Here, *W*_0_ is the initial area of the wound, and *W*_1_ is the area of the wound on the day of the study.

### 4.7. Statistical Analysis

The experiments were conducted with a minimum of three repetitions, and the average values, along with their corresponding standard deviations, were described (mean ± SD). One-way ANOVA and Mann–Whitney U tests were employed to compare groups, considering a significance level of *p* < 0.05. Statistical analysis and graph generation were performed using Excel and SPSS (V. 16.0) software.

## Figures and Tables

**Table 1:** Details of different electrospun scaffolds to select the proper blend ratio.

PCL/CS Ratio	Average Size ± (SD) nm	Bead Formation	Spinnability of the Solution
50:50	212 ± 25	High	Unsuccessful
60:40	123 ± 24	Rare	Successful
70:30	104 ± 21	Rare	Unsuccessful

**Table 2:** Toxicity% by the MTT assay: (N.C) negative control, (a) PCL/CS, (b) PCL/CS-Ag NPs, (c) PCL/CS-BRO, and (d) PCL/CS-BRO-Ag NPs, (P.C) positive control after 24 h.

Samples	N.C	a	b	c	d	P.C
Toxicity %	0.10 ± 0.02	40.49 ± 3.21	12.89 ± 1.57	18.79 ±1.23	10.44 ± 1.17	88.98 ± 5.27

## Data Availability

Data is available upon request.
